# Design and Analysis of Two Piezoelectric Cymbal Transducers with Metal Ring and Add Mass

**DOI:** 10.3390/s19010137

**Published:** 2019-01-02

**Authors:** Wenjie Wang, Weihao Shi, Peter Thomas, Mingsui Yang

**Affiliations:** 1 Fluid and Acoustic Engineering Laboratory, Beihang University, Beijing 100191, China; wangwenjie@buaa.edu.cn (W.W.); ShiWH@buaa.edu.cn (W.S.); 2 School of Engineering, University of Warwick, Coventry CV4 7AL, UK; P.J.Thomas@warwick.ac.uk; 3 AECC Shenyang Engine Research Institute, Shenyang 110015, China

**Keywords:** piezoelectric cymbal transducer, multilayer cavity structure, electrical admittance, resonance frequency

## Abstract

Based on traditional sandwich structure, two piezoelectric transducers were designed to meet the strict underwater application backgrounds such as high pressure, corrosion resistance, and high strength. Both transducers integrated most of previous researches while one transducer has a multilayer cavity structure which is different from the other structure and previous transducer structures. After a detailed simulation analysis of every structural parameter, key parameters were pointed out to have an obvious influence on its performance. Then, two models were constructed and compared with chosen sets of geometry parameters and manufacturing process, which can also provide a reference for low-frequency transducer design. The simulation results and experimental results of our transducers show a good consistency which indicates the cavity structure can reduce the resonance frequency.

## 1. Introduction

Due to the special nature of underwater environments and their associated conditions strict applied requirements for sensors and actuators exist such as high pressure, wide frequency band, and corrosion resistance. In the recent years, piezoelectric materials, including the ceramics ‘lead zirconate titanate’ (PZT), ‘lead titanate’ (PT), and ‘lead magnesium niobate’ (PMN) [1,2] and polymers ‘polyviylidene fluoride’ (PVDF) and ‘polyvinylidene fluoride trifluoroethylene’ P(VDF-TrFE) [3,4], were widely used for underwater transducers as active materials.

The piezoelectric ceramics can detect and show a very small mechanical vibration through its electrical signals, which has been widely used in sonar detection, household appliances, weather detection, telemetry environmental protection, and other practical occasions [5], because the piezoelectric ceramics are sensitive to external forces. Meanwhile, piezoelectric materials can be used for biomedical field [6,7,8]. Park has constructed the cymbal array as a practical ultrasound system for noninvasive transdermal insulin delivery for diabetes management [9]. Moreover, recent research on low frequency sensors based on PVDF and cymbal can be used to improve transdermal drug transport and achieve a more suitable therapeutic approach [10,11].

**Figure 1:**
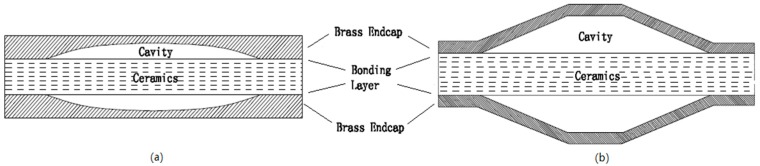
Moonie (**a**) and Cymbal (**b**) [15].

In the late 1980s, the university of Pennsylvania proposed two types of piezoelectric structures they referred to as “Moonie” [12,13,14] and “Cymbal” [15,16,17,18] with reference to the endcap shapes in Figure 1. These two active transducers have the same sandwich structure. The sandwich structure was connected by means of a thin layer of glue which decreased its efficiency [19,20]. The slotted endcap [21,22,23] was designed to reduce structure fatigue because arising from stress concentrations and improve its conversion coefficient. However, this complex endcap represents a big challenge as regards manufacture and construction. It is obvious that it is essentially impossible to use this slotted structure underwater and at high pressure. 

Uzgur [24] concluded that cavity depth will influence its maximum pressure. In order to improve the maximum pressure, Zhang [25,26,27] designed a novel piezoelectric transducer with a piezoelectric ceramic ring and another piezoelectric transducer with two piezoelectric ceramics. Then, a screw was welded to the endcap to lower the resonance frequency [17].

It is obvious that previous structure with bonding layer has a low strength and efficiency especially in deep water. In this paper, based on traditional structure two piezoelectric transducers names piezoelectric transducer A (PTA) and piezoelectric transducer B (PTB) as were reported. PTA adopted and integrated most of previous effective methods such as metal ring, add mass and none glue layer. PTB was designed with a multilayer cavity structure and compared with PTA. In these two transducers, optimizations of metal ring and add mass were adopted to protect the ceramic, avoid corrosion, and increase its strength. Every structural parameter of PTA was simulated and evaluated to investigate its influence. Section 2 illustrates the theoretical principle and physical mechanism in simulations. In Section 3, experimental arrangements and results are introduced and discussed. Then, the conclusions are summarized and displayed in Section 4.

## 2. Numerical Simulation

### 2.1. Theoretical Principle

**Figure 2:**
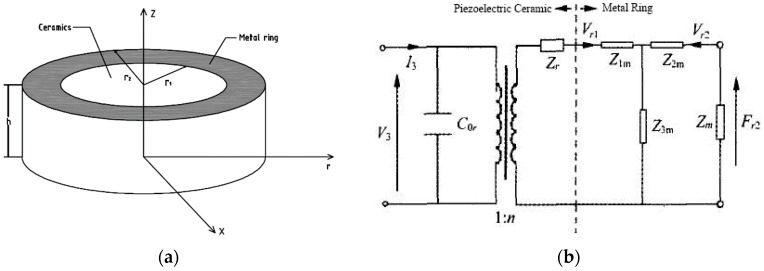
Key part and its electromechanical equivalent circuit. (**a**) Key part in the cylindrical coordinate system. (**b**) Electromechanical equivalent circuit.

When the piezoelectric ceramics was embedded in a brass ring, all parameters in this whole system were displayed in a cylindrical coordinate system in Figure 2a. The electromechanical equivalent circuit including piezoelectric ceramics and metal ring was illustrated in Figure 2b.

In Figure 2, *r*_1_ and *r*_2_ are inner radius and outer radius of the metal ring. *v_r_* is vibration velocity. *h* is the thickness of metal ring. *F_r_*_2_ and *F_r_*_3_ are forces act on inner surface and outer surface. *Z_m_* is the impedance of metal endcaps and screws. *Z*_1*m*_, *Z*_2*m*_, and *Z*_3*m*_ are equivalent mechanical impedance of metal ring. In the three-dimensional coordinate system, the ring vibration is described by

(1) $$\frac{\partial^{2}\xi_{r}}{\partial t^{2}}=V_{r}^{2}\left(\frac{\partial^{2}\xi_{r}}{\partial r^{2}}+\frac{1}{r}\frac{\partial \xi_{r}}{\partial r}-\frac{\xi_{r}}{r^{2}}\right)\;$$

where, *V* is the sound velocity of the radial vibration. 

The boundary conditions of inner surface and outer surface in the metal ring are

(2) $$\{\begin{array}{c} r=r_{1},v_{r}=v_{r2}\\ r=r_{2},v_{r}=-v_{r3} \end{array}$$

As for the brass ring, the force can be expressed.

(3) $$T_{r}=\frac{Ek}{1-v^{2}}\left\{A\left[J_{0}\left(kr\right)-\frac{\left(1-v\right)J_{1}\left(kr\right)}{kr}\right]+B\left[Y_{0}\left(kr\right)-\frac{\left(1-v\right)Y_{1}\left(kr\right)}{kr}\right]\right\}e^{j\omega t}$$

where, *A* and *B* are calculated from Equation (2). With the boundary conditions

(4) $$\{\begin{array}{c} r=r_{1},F_{r}=T_{r}S=-F_{r2}\\ r=r_{2},F_{r}=T_{r}S=-F_{r3} \end{array}$$

Therefore, the force can be expressed as

(5) $$F_{r2}=\left(Z_{1m}+Z_{3m}\right)v_{r2}+Z_{3m}v_{r3}$$

(6) $$F_{r3}=\left(Z_{2m}+Z_{3m}\right)v_{r3}+Z_{3m}v_{r2}$$

In the actual applications, all the connections between the metal ring, the ceramic and the endcaps are rigid. The exact theoretical solution of endcaps vibrations is hard to be resolved because of complex geometry and flextensions. However, according to velocity continuity, force continuity and rigid connection between ceramic and brass ring, the electromechanical equivalent circuit can be displayed when the endcap resistance was assumed as *Z_m_*.

In the electromechanical equivalent circuit, the following expressions [28] can be obtained according to Kirchhoff circuit laws.

(7) $$I_{3}=j\omega C_{0r}V_{3}+nV_{r1}$$

(8) $$nV_{3}=V_{r1}\left(Z_{r}+Z_{1m}\right)+Z_{3m}\left(Z_{r1}+Z_{r2}\right)$$

(9) $$F_{r3}=V_{r2}Z_{2m}+Z_{3m}\left(V_{r1}+V_{r2}\right)$$

(10) $$F_{r3}=-V_{r2}Z_{m}$$

Therefore, the electrical admittance of piezoelectric transducer can be expressed from Equations (7)–(10).

(11) $$Y=\frac{I}{U}=G+jB=j\omega C_{0r}+\frac{n^{2}}{Z_{r}+Z_{1m}+\frac{Z_{3m}\left(Z_{2m}+Z_{m}\right)}{Z_{m}+Z_{2m}+Z_{3m}}}$$

When the electrical impedance is a minimum, the resonance frequency can be obtained. Otherwise, the antiresonance frequency will be solved with the maximum impedance.

### 2.2. Structural Parameter Analysis

A piezoelectric transducer (PTA) was designed while metal ring and add mass were adopted at the same time. Every structural parameter of this integrated design will be simulated and evaluated to find out key parameters which can influence its performance obviously in this section. 

**Figure 3:**
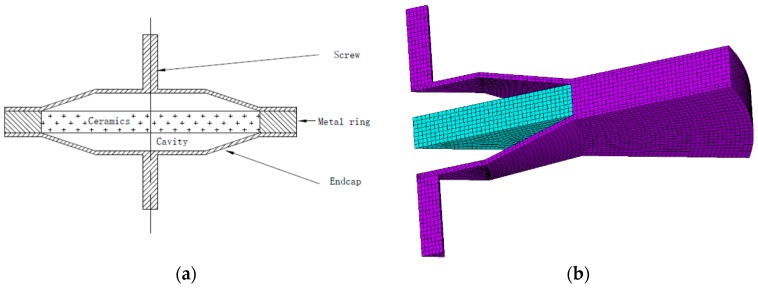
PTA structure and its mesh. (**a**) PTA geometry. (**b**) PTA mesh in simulations.

Based on the sandwich structure, a brass ring was connected to the piezoelectric ceramics by heat expansion and contraction instead of glue layer to increase its strength. Meanwhile, screws were welded to the brass endcaps to lower its resonance frequency. The whole structure and its mesh were displayed in Figure 3.

All materials used include brass and piezoelectric ceramics. The piezoelectric ceramics can be classified into two types (PZT 4 and PZT 5). Piezoelectric performance parameters of these two types are usually constant values. The material performance parameters of our PZT 4 are summarize in Table 1 where the obvious difference between PZT 4 and PZT 5 is the relative dielectric [29,30].

In the numerical simulation, all the performance parameters of piezoelectric ceramic, brass ring, metal screws, and endcaps were assumed as uniform without any local special or specific structure. The electrode influence was neglected for simplified calculation model and solving efficiency when the simulation accuracy can be guaranteed. 1/12 of the whole structure (30° in the circumferential direction) is meshed for calculations because the structure is axially symmetric.

All the simulations are based on the software ANSYS since no theoretical solution can be expressed with such a complex endcap. SOLID 45 is used for the three-dimensional modeling of solid structures. The element is defined by eight nodes having three degrees of freedom at each node: translations in the nodal *x*, *y*, and *z* directions. The piezoelectric ceramic adopted SOLID 5 which has a three-dimensional magnetic, thermal, electric, piezoelectric, and structural field capability with limited coupling between the fields. The element has eight nodes with up to six degrees of freedom at each node.

The boundary conditions include electrical conditions and mechanical conditions. The symmetric boundary condition was set on the radial boundary surfaces. Meanwhile, the open circuit electrical boundary condition is used on the two radial sections of the piezoelectric ceramic, and the harmonic alternating excitation electric field signal on the two electrode surfaces of the piezoelectric ceramic.

It is obvious that structural parameters will influence its efficiency. However, every parameter should be evaluated how much it can affect the resonance frequency. Therefore, we simulate and analyze with different structure parameters such as ceramic thickness *t2*, endcap thickness *t1*, screw length *t3*, screw radius *r0*, cavity radius *r1*, ceramic radius *r2*, outer radius of metal ring *r3* and cavity depth *h1* in Figure 2 where these basic structure parameters were illustrated and displayed. 

**Figure 4:**
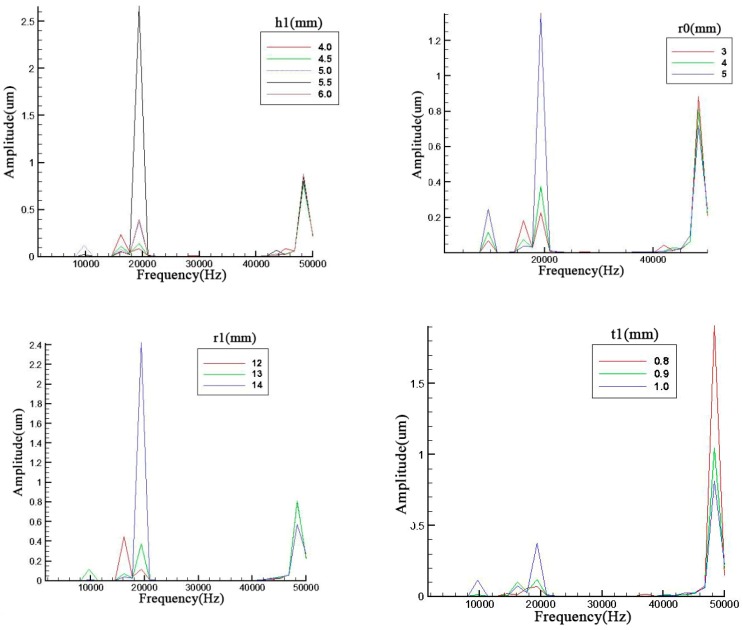
Parameter analysis of *h1*, *r0*, *r1*, and *t1*.

Parameter influences were displayed in Figure 4 and all the trends are basically the same. It can be seen that all these geometry parameters can primarily affect the amplitude of the resonance frequency especially for the third order. Meanwhile, the second and third resonance frequencies have an obvious opposite trend. The odd and even orders have the same trend.

With an increase of the cavity depth, the cavity diameter and the screw diameter, the resonance frequency decreases slightly and the amplitude of the odd orders also display a decrease. As for the metal endcap, the endcap thickness will prominently influence the 4th order resonance frequency.

**Figure 5:**
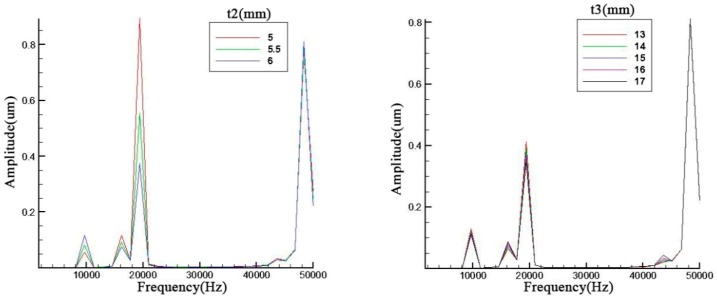
Parameter analysis of *t*2 and *t*3.

**Figure 6:**
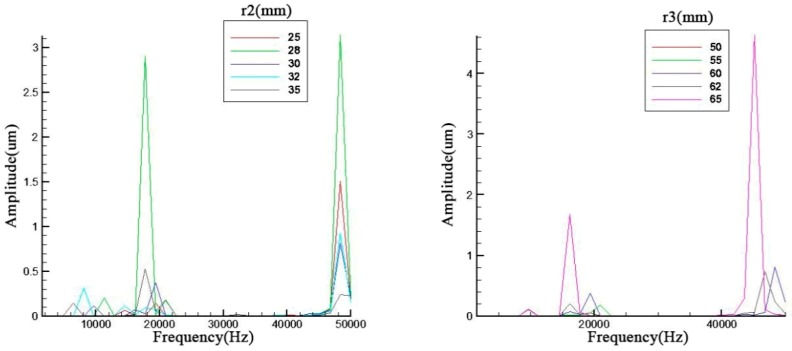
Parameter analysis of *r*2 and *r*3.

In Figure 5, the ceramic thickness and brass screw length are discussed. contrary to the previous discussions relating to figures *x* and *y*, the second and third have the same trend which is the same occasion for the first and fourth in the left figure of Figure 6. However, the metal screw has a small effect on the result. The fluctuation of resonance frequency was in a narrow scope because the add mass is too small compared with the whole piezoelectric transducer.

The central devices of a piezoelectric transducer are piezoelectric ceramic and brass ring. Those structure parameters should have a stronger effect on the resonance frequency. The diameter of inner circle in the metal ring is also the ceramic diameter. The ceramic diameter can change the resonance frequency as evident from Figure 6. Meanwhile, the diameter of outer circle has a more obvious effect on the result. The difference is that it is not a linear trend. There is a maximum in the amplitude of resonance frequency. 

**Figure 7:**
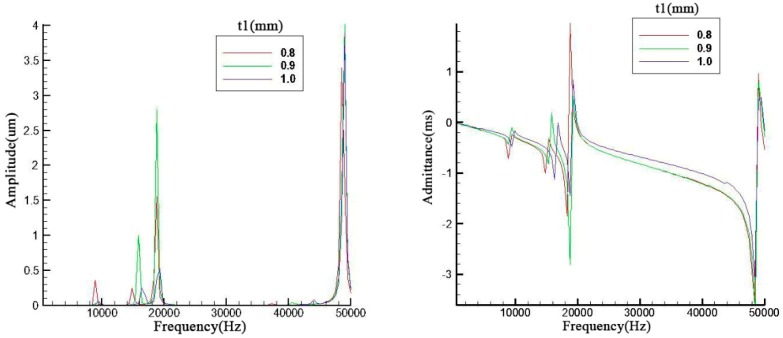
Simulation results with more analysis points.

From these simulation results, it can be seen that some geometry parameters such as endcap thickness may have a little influence on its resonance frequency and amplitudes. This may cause by the influence occurred between two analysis frequency points. Therefore, after the analysis points of endcap thickness were tripled, the results are shown in Figure 7. The results indicate that the endcap thickness can change the resonance frequency more obviously when the number of analysis points was improved. However, the aim in this paper was to find out the most prominent parameter which can effectively change results. If the change occurred in a narrow band, it is not the prominent parameter.

## 3. Comparisons and Analysis of Two Transducers

### 3.1. Simulations of PTA and PTB

**Figure 8:**
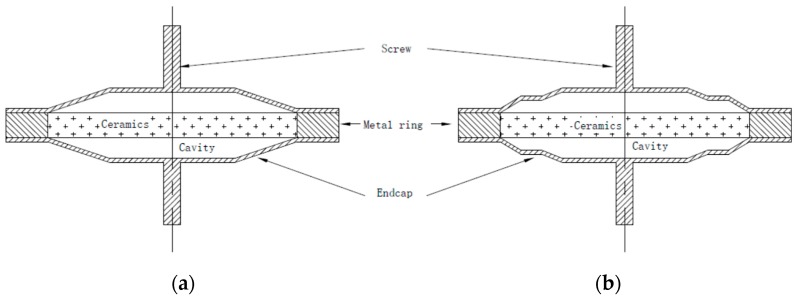
Structures of PTA and PTB. (**a**) PTA structure. (**b**) PTB structure.

According to the parameter analysis in Section 2, we choose a set of structure parameters to construct PTA and similar parameters for PTB. These two piezoelectric transducers were displayed in Figure 8.

In order to compare these two piezoelectric transducers, most of structural parameters were the same, excluding cavity depth. Meanwhile, the total depth of multilayer cavity in the right section of Figure 8 equaled to cavity depth in the left section of Figure 8. All the structural parameters were introduced in Table 2.

**Figure 9:**
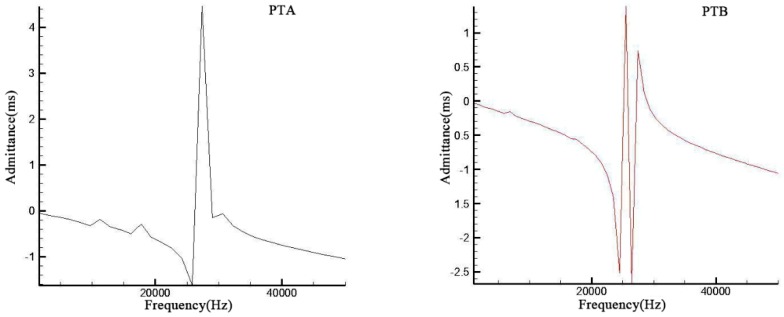

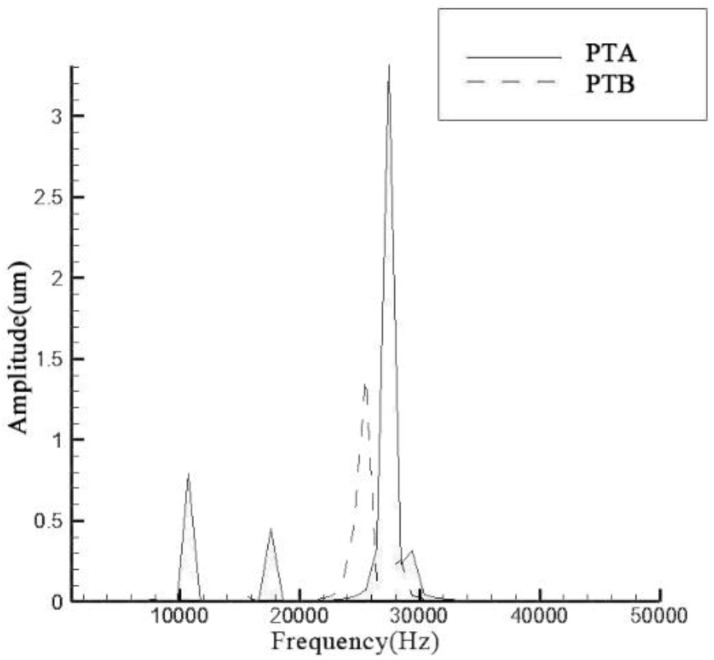
Simulation results of two transducers.

Based on the same boundary conditions and external load in Section 2, the admittances and resonance frequencies of these two transducers were obtained. The biggest resonant frequencies at third order are 27.45 kHz and 25.49 kHz respectively in Figure 9.

It is obvious in Figure 9 that all the amplitudes and admittances of PTB at every order were much lower than those of PTA while PTB have a lower resonance frequency due to multilayer cavity structure. This indicates that more cavity structures may decrease the resonance frequency. Compared with the third resonance frequency, both amplitudes and admittances of the first two orders were much lower in these two transducers. Meanwhile, amplitudes of the first two orders of PTB even cannot be displayed under the same range due to their too small values which has an indirect display in the admittance figures.

### 3.2. Experiment Results

**Figure 10:**
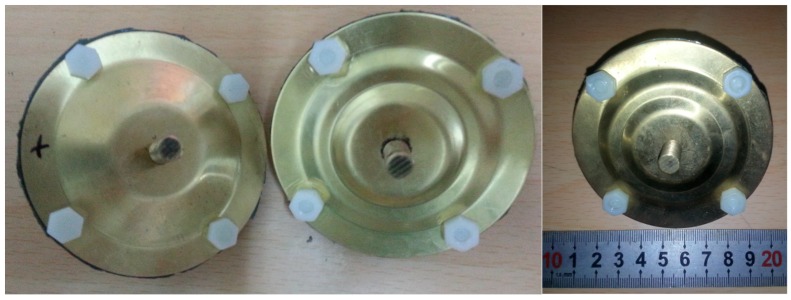
Two constructed transducers.

According to the simulation results and structure parameter analysis, two sets of structure parameters were adopted to construct the transducers shown in Figure 10. Ceramics were put into a brass ring by heat expansion and contraction with the insulating paper between them. Endcaps were connected with the brass ring by insulated screws. In order to measure the resonance frequency by external electric field, fine wires were held between endcaps and ceramic. Two brass screws were held to endcaps as add mass.

**Figure 11:**
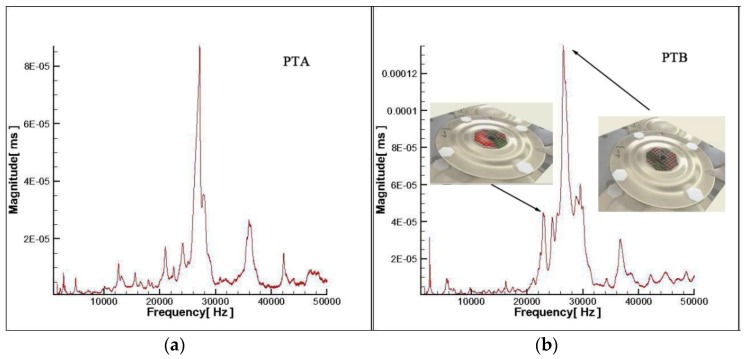
Experimental results. (**a**) PTA. (**b**) PTB.

Based on the parameter analysis results, two transducers were constructed with two sets of parameters. The transducers were measured by laser vibrometer (PSV-400). The measurement point number on the top surface of piezoelectric transducer is 100 and the frequency band is 1–50 kHz. Therefore, the figure of vibration velocity vs. frequency was shown in Figure 11. 

The biggest resonance frequencies in Figure 11 are 27.14 kHz and 26.48 kHz respectively which were consistent with simulation results. The tiny deviation may be due to mass loss from threaded holes. It can be found that the biggest resonance frequency of PTB in Figure 11 has a wider bandwidth while two vibration modes in the resonance bandwidth were illustrated. The resonance bandwidth of PTB was from 25.766 to 27.906 kHz while the biggest resonance frequency was 26.48 kHz.

## 4. Conclusions

Structural parameters were simulated in detail and evaluated to influence its performance. Almost every geometry parameter of PTA was simulated to find out the most prominent parameter which can influence its performance. The simulation results indicate that the dimeters of ceramic and brass ring can change the resonance frequency obviously. Especially, some parameters have a such a minor influence that the influence may be neglected in an analysis step. Other parameters can mainly affect the amplitudes. The influence trend of every parameter was introduced.

In the manufacturing process, heat expansion and contraction was adopted to avoid low intensity of glue layer. However, this technological means will cause an uncertain prestress between ceramics and metal ring which is an error factor. Insulation measures can ensure steady operation of the system and improve the structural stability.

Two transducers were simulated and measured separately. The experimental results of PTA and PTB were consistent with simulation results. Compared with the PTA, the results of PTB show that more cavity structure can decrease the resonance frequency. It can provide a reference for further transducer design.

## Figures and Tables

**Table 1:** Material performance parameters.

Ceramics	Piezoelectric Stress (C/m^2^)			Relative Dielectric			Elastic Stiffness (10^10^N/m^2^)					
	$e_{31}$	$e_{33}$	$e_{15}$	$\varepsilon_{11r}$	$\varepsilon_{22r}$	$\varepsilon_{33r}$	$c_{11}$	$c_{12}$	$c_{13}$	$c_{33}$	$c_{44}$	$c_{66}$
PZT5	−5.4	15.8	12.3	916	830	830	12.1	7.54	7.52	11.1	2.11	2.26
PZT4	−5.2	15.1	12.7	730	730	635	14.9	7.78	7.43	11.5	2.56	4.06

**Table 2:** Structural parameters of two piezoelectric transducers (mm).

Transducer Name	Screw Radius	Screw Length	Endcap Radius			Endcap Thickness	Ceramic Radius	Ceramic Thickness	Ring Radius	Cavity Depth	
PTA	4	15	**15**			1	30	6	40	**5**	
PTB	4	15	**15**	**20**	**25**	1	30	6	40	**2**	**3**
